# The Different Mechanisms of Cancer Drug Resistance: A Brief Review

**DOI:** 10.15171/apb.2017.041

**Published:** 2017-09-25

**Authors:** Behzad Mansoori, Ali Mohammadi, Sadaf Davudian, Solmaz Shirjang, Behzad Baradaran

**Affiliations:** ^1^Immunology Research Center, Tabriz University of Medical Sciences, Tabriz, Iran.; ^2^Student Research Committee, Tabriz University of Medical Sciences, Tabriz, Iran.

**Keywords:** Drug resistance, Cancer, Multi-drug resistance, microRNA, Epigenetic, Cell death inhibiting

## Abstract

Anticancer drugs resistance is a complex process that arises from altering in the drug targets. Advances in the DNA microarray, proteomics technology and the development of targeted therapies provide the new strategies to overcome the drug resistance. Although a design of the new chemotherapy agents is growing quickly, effective chemotherapy agent has not been discovered against the advanced stage of cancer (such as invasion and metastasis). The cancer cell resistance against the anticancer agents can be due to many factors such as the individual’s genetic differences, especially in tumoral somatic cells. Also, the cancer drug resistance is acquired, the drug resistance can be occurred by different mechanisms, including multi-drug resistance, cell death inhibiting (apoptosis suppression), altering in the drug metabolism, epigenetic and drug targets, enhancing DNA repair and gene amplification. In this review, we outlined the mechanisms of cancer drug resistance and in following, the treatment failures by common chemotherapy agents in the different type of cancers.

## Introduction

By providing advances in the cancer research, our knowledge of the cancer biological characteristics is updating every day. Cancer causes the uncontrolled growth of abnormal cells and dynamic altering in the genome (which cause cancerous features in normal cells).^1^ The cancer progression impairs the normal biological process of healthy cells which achieved by the invasion of nearby tissues and metastasize to distant tissues.^2^

**Figure 1:**
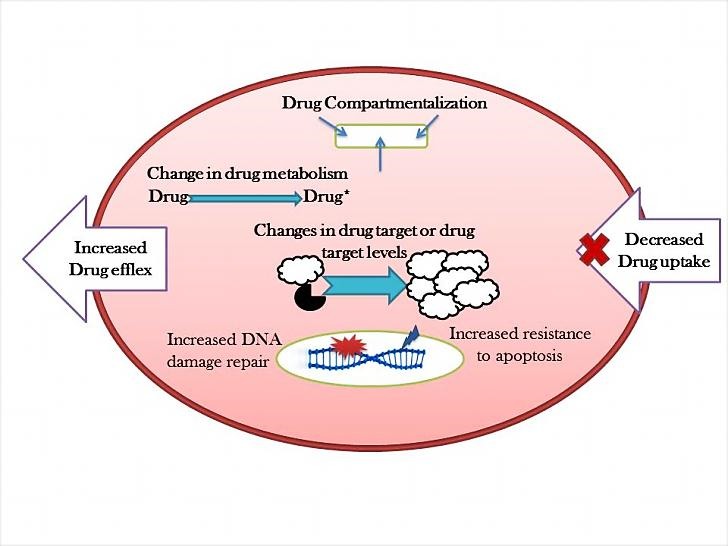
**The mechanisms of drug resistance in the cancer cells.** Cancer cells will become resistant to drugs by the mechanisms such as the inactivation of the drug, multi-drug resistance, cell death inhibition (apoptosis suppression), altering in the drug metabolism, epigenetic changing, changes in the drug targets, enhances DNA-repair and target gene amplification.

In addition to,‏‏ common cancer treatments such as surgery, radiation therapy, chemotherapy, combination therapy and laser therapy; the selective therapies are based on the better conception of the biology and molecular genetics in the tumor progression used for the promising treatments.^3^ Todays, despite these advances, the promising option for cancer treatment is chemotherapy. Currently, 90% of failures in the chemotherapy are during the invasion and metastasis of cancers related to drug resistance. In the chemotherapy, by following the administration of a certain drug, a large number of patient tumor cells become resistant to the drug. So, the drug resistance appears as a serious problem in the field of cancer.^4^ There are many problems in the cancer therapy, such as cytotoxic agents resistance and toxic chemotherapy.^5^ The novel cancer treatments by studying on the molecular targets of oncogenes, tumor suppressor genes and RNA interference (RNAi) are expanded.^6^ The purposes of these therapies include 1. The kinases inhibition that involved in the cell proliferation, 2. Improving the rapid immune responses in cancer, 3. Specializing the medications, 4. Drug delivery into cancer cells and 5. reducing the side effects of anticancer drugs, etc.^7^ There are several mechanisms including inactivation of the drug, multi-drug resistance, inhibiting cell death (apoptosis suppression), changes in drug metabolism, epigenetic and drug targets, enhance DNA repair and gene amplification that cause the resistance to the chemotherapy (Figure 1).

In this review, we outlined the different mechanisms involved in cancer drug resistance and glance over the reason of treatment failures by common chemotherapeutic agents in cancer, and finally, we proposed the novel strategies to overcome the cancer drug resistance.

## Intrinsic and extrinsic factors in drug resistance

### Tumor heterogeneity

Intra-tumor heterogeneity can be observed at many different cancer levels and may be assignable to a number of different factors that primarily occur at the cellular level. This means, the natural generation of variants form which are considered by various genetic, epigenetic, transcriptomic and proteomic properties. The genotypic changes include: mutations, gene amplifications, deletions, chromosomal rearrangements, transposition of the genetic elements, translocations and microRNA alteration. Genomic instability generates a great level of intercellular genetic heterogeneity in cancer. Epigenetic factors including miRNA, transcriptomic and proteomic heterogeneity may rise due to primary genotypic variations, but can also reflect cell cycle stage, stochastic variations between cells, or hierarchical organization of cells according to the cancer stem cell theory.^8-12^ These alterations known as intrinsic factors cause tumor heterogeneity. Extrinsic factors include pH, hypoxia, and paracrine signaling interactions with stromal and other tumor cells.^13,14^

These factors change, increase, or diminish gene products which directly are involved in the generation of drug resistance and poor prognosis.

### Tumor microenvironment

Growing evidence supports the important role of tumor microenvironment in drug resistance discussion as the main reason for the relapse and incurability of various cancers. The tumor microenvironment involves normal stromal cells (SC), extracellular matrix (ECM), and several soluble factors include cytokines and growth factors. Tumor-tumor cell communication, tumor-stromal cell communication, as well as tumor-ECM interface, all contribute to direct cell interaction mediated by drug resistance.^15^ Moreover, growth factor (GF), cytokines produced in the tumor microenvironment provide additional signals for tumor cell growth and survival. Environment mediated-drug resistance (EM-DR) could be well thought-out as the whole of cell adhesion mediated drug resistance (CAM-DR) and soluble factor -mediated drug resistance (SM-DR) products including VEGF (vascular endothelial growth factor); bFGF (basic fibroblast growth factor); SDF-1 (stromal cell-derived factor- 1); IL-6 (interleukin-6); NO (nitric oxide); IL-3 (interleukin-3), G-CSF (granulocyte colony stimulating factor); M-CSF (macrophage colony stimulating factor); GM-CSF (granulocyte-macrophage colony stimulating factor); TNF super family members BAFF (B cell-activating factor of the TNF family) and APRIL (a proliferation-inducing ligand); and many others by the tumor cell interaction.^15-17^

### Cancer stem cells

Cancer stem-cell populations have been detected in a variety of hematopoietic and solid tumors, and might be the cell of origin of hematopoietic and solid tumors. Although chemotherapy impairs an enormous number of cells in a tumor, but it is understood that the chemotherapy agents are removed from cancer stem cells with the special mechanisms, which might be an important for drug resistance, for instance, overexpression of the ATP-binding cassette (ABC), drug transporters such as ABCB1, which encodes P-glycoprotein, and the ABCG2, which was originally identified in mitoxantrone resistant cells have been shown to keep cancer stem cells away from chemotherapeutic agents. Cancer stem cells share several of normal stem cells possession that provides for a long lifetime, including the relative silence, resistance to drugs and toxins through the expression of drug efflux transporters, an active DNA-repair capacity and a resistance to apoptosis, vascular niche, dormancy, hypoxic stability and enhance activity of repair enzymes.^18-20^ Following the cancer cells features mentioned above, these cells remain stable in the patients recovering seemingly or metastasize to distant organs and cause the cancer recurrence. So, the identifying and eliminating these small populations of cancer cells is such a significant help to eliminate the drug resistance.

## Inactivation of the anticancer drugs

The anticancer drugs efficiency and their activity are dependent on the complex mechanisms. The interaction between drugs and different types of proteins (in vivo) can alter the molecular characteristics of drugs and ultimately activate them. Cancer cells become resistant by reducing the activity of drugs.^21^ The acute myeloid leukemia (AML) treatment with cytarabine (AraC) (an anti-cancer drug nucleotide after multiple phosphorylations can be converted to cytarabine triphosphate (AraC-triphosphate)‏( is an example of this context. AraC has no effect on the cancer cells at the first step, but its phosphorylated form is lethal to cells and damages them.^22^ Down-regulation or mutations in the proteins and enzymes involving in this pathway (phosphorylation reactions) reduce the AraC activity and it causes drug-resistant cancer cells to AraC.^23^

Another important example of anti-cancer drugs is glutathione S-transferase family (GST) that has three large super families such as cytosolic, mitochondrial and microsomal-also, known as MAPEG proteins. This group of the enzyme has a major role in the detoxification of drugs, ionizing molecules and electron compounds in the cell. GST enzymes increase the drug resistance in cancer cells directly by the detoxification of anti-cancer drug or indirectly by the mitogen-activated protein kinase (MAPK) pathway inhibition in the RAS-MAPK path. The increased expression of GST in the cancer cells and follow the increasing levels in the detoxification of anticancer drugs, reduce the damages and lethality of these drugs on the cancer cells. Also, it is associated with increasing the resistance to apoptosis, induced by various stimuli.

## Multi-drug resistance (MDR)

Multi-drug resistance (MDR) in the cancer chemotherapy has been pointed out as the ability of cancer cells to survive against a wide range of anti-cancer drugs ^22^. MDR mechanism may be developed by increased release of the drug outside the cells. So the drug absorption is reduced in these cells.^24^

### Increasing the release of drugs outside the cell

**Figure 2:**
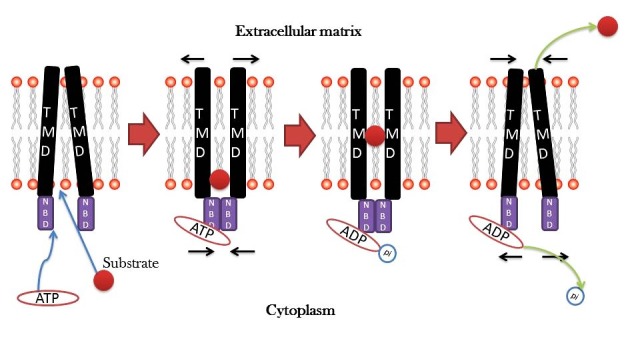
**The drug releasing mechanism through ABC transporters outside the cell.** After binding the substance (drug), ATP is hydrolyzed, the phosphate group is released and the energy of ATP hydrolysis leads to change in the ABC conformational, then the drug is released into the extracellular space.

There is a family of ATP-dependent transporters which involved in the transporting of the nutrients and other molecules across the membrane. The ABC transporters are composed of two cytoplasmic domains that bind to ATP known as ATP-binding cassette (ABC) and two transmembrane domains(TMDs).^24^ ABC Family has three members, including 1. P-glycoprotein (PGP), 2. multi-drug Resistance-associated Protein 1 (MRP1) and 3. Breast Cancer Resistance Protein (BCRP/ABCG2).^25^ P-Glycoprotein (P-gp) which is a multidrug membrane transporter that normally known as a pump for the moving chloride out of the cells and can bind to the variety of chemotherapy agents, including Doxorubicin, Vinblastine and Taxol, following binding ATP hydrolyzed and then the structure of P-gp has been altered. As a result, the agent releases to the extracellular space. Following the second ATP hydrolysis, the transporter returns its basic structure and is able to release the drug outside of the cell (Figure 2).^26,27^

### Reducing the absorption of the drugs

The absorption of the anticancer agent into the tumoral cells can occur by passive transfer (e.g., doxorubicin and vinblastine), facilitate diffusion, activate the transport (for example, nucleoside analogs).^28^ The cytotoxic agents are able to enter the cells via direction of the concentration gradient by the three ABC transporter molecules which were mentioned above. But the absorption of the drug into the cells via direction of a high concentration gradient occurs only through active transport.^29^ Most of the membranes transporters belong to solute carrier SLC transporters (transports minerals, vitamins and etc). Reducing the absorption of the drugs can occur at two main ways: 1. reducing the tendency to drugs binding and/or 2. Reducing the numbers of transporters. Some of the agents use the specific transporters to enter the cells.^30^ Mutations in these transporters inhibit them and reduce the absorption of the drugs. The resistance to Methotrexate is occurred usually by the human folate carrier's (hRFC) gene mutation in the patients with acute lymphoblastic leukemia (ALL). The mutation of G point at nucleotide 133 and the substitution of lysine by glutamic acid in the first transmembrane domain of hRFC protein reduces the tendency of the drugs to bind the transporter.^31^

## Inhibition of the cell death (apoptosis pathway blocking)

The cell death is mediated by the three important events such as necrosis, apoptosis, and autophagy. However, these processes differ from each other in their biological characteristics. All of them facilitate the cell death. Apoptosis occurs through both internal and external pathways. On its external pathway, the ligands and cell death receptors such as FAS, TNF-R, linker proteins, caspases-3, -6, -7 and -8 are involved. As a result, the proteolysis of actin protein, and nuclear lamin proteins occurs in the external pathway and ultimately leads to cell apoptosis.^32^ In the internal pathway performed in the mitochondria such as Bcl2, AKT act as the anti-apoptotic proteins, and Bax, Bak and caspase-9 act as the pre-apoptotic proteins. The up-regulation of the anti-apoptotic genes (Bcl2, AKT and etc) and down- regulation of pre-apoptotic genes (Bax, Bcl_xl_ and etc) in tumor cells are associated with increased resistance to chemotherapy.^33^ Also, the drug resistance occurred by the mutations in the p53 gene, can induce apoptosis in the cell stress and DNA damaging. These mutations could impair the connection between DNA damage (which caused by chemotherapeutic agents) and the activation of apoptosis.^34^

## Changing the drug metabolism

Chemotherapeutic agent metabolisms can be occurred by enzymes. Enzymes are the most important factors for determining the agent concentration, the inner and outer of the cells. Reactions to the agents such as oxidation, reduction and hydrolysis which are known as phase I reactions, and the consumption and conversion which are known as a phase II reactions play an important role in protecting normal cells against toxic agents. These reactions reduce the drug resistance in the cancer cells via two manners including 1. reducing the activation of pro-drugs (reduced the activity of some enzymes) and 2. increasing the drug inactivation (increased activity of some enzymes). One of the important examples in the phase I reactions which managed with cells is the detoxification done by cytochrome P450.^35^ The drug resistance in the breast cancer with increasing the activity of cytochrome P450 has been reported, also the enhancing of the cytochrome P450 resulted in the docetaxel inactivation.^36^ On the same hand, along with reducing the activity of this enzyme, the better response to the treatment has been observed. The phase II reaction of the drug (consumption phase) which was converted to glucuronic acid, sulfate and glutathione, these reduce the drug activity and dispose of its electrophilic toxicity.^37^ Increasing the production of glutathione and the detoxification occurred by glutathione transferases which play an important role in the resistance to many alkylating agents and platinum-based anticancer drugs such as cisplatin and doxorubicin.^37^

## Changing the chemotherapeutic agents targets

The effect of chemotherapeutic agents could have been depended on the modifications such as the mutations and changes in the expression levels of their targets. These types of modifications in the agent targets will lead to drug resistance, eventually.^38^ For example, the topoisomerase enzymes are responsible for opening the compaction in the structure of DNA during the replication.^39^ Doxorubicin, mainly used for the treatment of the solid tumors (such as breast cancer and lung tumors), originates from anthracycline fungus antibiotic could inhibit Topoisomerase II. Cancer cells with the mutations in topoisomerase II alter the purpose of the mentioned drug.^40^

One of the most common drug resistances, due to the secondary mutations and also it is known as the major mechanism which causes drug resistance and changing in the drug targets, is the imatinib resistance in the chronic myelogenous leukemia (CML). In CML, a Philadelphia chromosome is formed by the translocation between 9 and 22 chromosomes occurred at the 3' end of ABL gene on the chromosome 9 and at the 5' end of BCR gene on the chromosome 22; (9q34; 22q11. 2) (9; 22) t (22) (Table 1).

**Table 1:** Disease and drug resistance mechanisms and pathways interruption

**Disease**	**Drug resistance Mechanism and pathways interruption**	**References**
CML	Resistance to imatinib BCR-ABL Mutations (9; 22) t (22)	^21,38^
Myeloproliferative disorders	JAK2	^41^
AML	GSK-3b activity adhesion and Wnt-pathway b-catenin expression SHIP mutations PI3-kinase/Akt activation	^42-44^
ALL	Increased Akt expression Regulation protein-1 and PI3K signaling PTEN mutation/deletion/inactivation	^45,46^
Other human neoplasia	involvement of the Ras/Raf/MEK/ERK, PI3K/ PTEN/Akt and Jak/STAT cascades AKT/PKB signaling Raf/MEK/ERK pathway PTEN	^1,46,47^

In a BCR-ABL translocation, involving the different parts of the two genes depending on which chromosomal breakpoints situation. The drug resistance processes are multifactorial. The point mutations and amino acid substitution in the kinase domain of BCR-ABL lead to altered structure in the proteins and prevent the proper binding of the drugs.

Up till now, approximately 70 different types of the mutations have been reported in the kinase domain of BCR-ABL.

The fifteen amino acids substitutions have been reported in the 80% to 90% of the mutations cause the resistance to imatinib. Most of the mutations (60-70%) occur in the 7 common locations, including Y253, E255, T315, M351, F359, H396, and G250T. Most of these mutations occur in the 4 hot spots of the kinase domain containing the A-Loop, C-Loop, P-Loop, and, ‘Drug Contact site', the last is the binding site of imatinib.

The differences in the resistance to Imatinib will depend on the types of mutations. For an example, the mutation of M351 causes the weak resistance to imatinib. So, we need the increasing dose of imatinib. If the mutations such as T315, V299L be detected, we need for using of the second-generation of drugs (dasatinib). Nilotinib, the second generation of imatinib, is usually used for Y253, E255 mutations in the P-Loop.

As a result, the point mutations (Missense) in CML in the kinase domain can change the conformational structure in the protein and then block the ATP-binding site of imatinib to its binding site. So, the protein is always active and the activity of tyrosine kinase is enhanced.^48-50^

## Enhancing the DNA repair

DNA repair is one of the well-known mechanisms of the drug resistance in cancer field. The chemotherapeutic agents damage directly or/and indirectly the cancer cells DNA, so, there are mechanisms that can repair the damage of DNA. For example, platinum-based agents such as cisplatin cause DNA damage which leads to the apoptosis of tumoral cells.^51^ The resistance to these agents occurs by the DNA repair systems, including nucleotide excision repair system (NER) and homologous recombination repair mechanisms (RRM) in the cancer cells. So, the efficiency of these agents is dependent on the inhibition of the DNA repair systems in the cancer cells. The inhibition of DNA repair systems sensitizes the cancer cells to these drugs and thus the effectiveness of the chemotherapy will increase. The defects in the DNA repair systems in the cancerous cells could be one of the therapeutic targets which can be possible by mutations and epigenetic silencing in these systems.^52^ The enhancement of DNA repair and alkyltransferase activity also cause the resistance to doxorubicin (alkylating agent).^53^

## Gene amplification

Gene amplification is a mechanism of the drug resistance in 10% of the cancers, especially in leukemias. Increasing the numbers of target genes by the gene amplification in some tumoral cells, including leukemia cause the drug resistance to Methotrexate.^54^ The cancer cells cause the drug resistance via providing the multiple copies of the Dihydrofolate reductase gene (could target enzyme of methotrexate). The gene amplification increases the copy numbers of the oncogenes per cells to several hundred folds. Finally, this mechanism cause to the production of larger amounts of the related oncoproteins.^55^ The sequences amplified in the cancer cells are detectable with additional small chromosomes called double chromosomes (DMs- double minute chromosome) or homogeneously staining regions-HSR in the final stages of malignancy.^56^

## Epigenetic altering caused drug resistance

One of the important mechanisms of the drug resistance in the cancer therapy is the epigenetic altering. There are two types of the epigenetic altering such as 1. methylation of DNA and 2. histone alterations.^57^ The DNA methylation is a major epigenetic phenomenon that occurs with the methylation of the cytosine by methyltransferase in 5’ carbon in the CpG islands (an upstream of the promoters). However, the methylation can occur throughout the genome in other positions.^58^ Acetylation and deacetylation of the specific lysine located at the terminal ends of histones and non-histone proteins performed by histone acetyltransferases (HATs) and histone deacetylases (HDACs) enzymes respectively. These enzymes alter the structure and composition of chromatins. The acetylation of lysine open the chromatin structure, and deacetylation of this unit (lysine) cause the chromatin compaction and the stability of them, these mechanisms regulate the gene expression.^50^ For example, the tumor suppressor genes often silenced by methylation, in contrast, the hyper-methylation of oncogenes induced their expression.^59^ Demethylation of multi-drug resistance gene (MDR1), in the cancer cell lines, leads to the acquisition of multi drug-resistant phenotype and reduces the accumulation of the anti-tumor drug inside the cancer cells. MDR1 is overexpressed in the premature myeloid cancer cells, but the mature myeloid cancer cells decrease the expression of MDR1.^60^

The epigenetic mechanism can also affect their DNA repair system. In the mismatch repair system several proteins including hMLH1, hMSH1 and etc. are involved. The mutations or hypermethylation in the promoter of following genes cause cancer. For example, the mutation or hypermethylation of hMLH1 gene can cause the colorectal cancer.^61^ 5-Aza-2'-deoxycytidine (decitabine; DAC) used for the inhibition of DNA methylation, which has no effect on the tumor growth, but it sensitizes the tumor to other drugs such as cisplatin and carboplatin.

Similarly, the demethylation of hMLH1 promoter gene by DAC and recovery of the mismatch repair system causes the colorectal cancer cells to become sensitive to 5-FU (fluorouracil -5).^62^ So the combination of epigenetic and conventional chemotherapeutic agents are effective in the treatment of resisted tumors and cancerous cells.^63^

## MicroRNA in cancer drug resistance

MicroRNAs (miRNAs) are ~22 nucleotide RNAs processed from RNA hairpin structures. MicroRNAs are much too short to code for protein and instead play important roles in regulating gene expression. They regulate most protein-coding genes, including important genes in cancer and especially in cancer drug resistance generation. There are three mechanisms involved in gene silencing with miRNA process: 1. Cleavage of the mRNA strand into two pieces, 2. Destabilization of the mRNA through shortening of its poly(A) tail and, 3. Less efficient translation of the mRNA into proteins by ribosomes.

Recent studies in miRNA profiling confirmed that these small molecules play an important role in the development of chemosensitivity or chemoresistance in different types of cancer (Table 2). miRNA might involve in all the drug resistance mechanisms which mentioned above. miRNAs could increase the efficacy of tumors to chemotherapy agent or it could avoid cancer drug resistance. Also, these small molecules could serve as a biomarker for prognosis and survival in response to chemotherapy.

**Table 2:** miRNAs involved in cancer drug resistance

**miRNA**	**Target**	**Tumor**	**Chemotherapy agent**	**Reference**
miR-7	MDR1	SCLC	Anthracyclines	^64^
miR-9	MDR1/ABCG2	Glioblastoma	Temozolomide	^65^
miR-17-5p	PTEN	Ovary	Paclitaxel	^66^
miR-21	PTEN, PDCD4	Breast	Trastuzumab	^67^
miR-25	ABCG2	Breast	Epirubicin	^68^
miR-103/107	P-gp	Gastric	Doxorubicin	^69^
miR-127	MDR1/MRP1	Glioma	Adriamycin	^70^
miR-129-5p	ABCB1	Gastric	Vincristinecisplatin 5-fluorouracil	^71^
miR-134	MRP1/ABCC1	Breast	Doxorubicin	^72^
miR-145	P-gp/ABCB1	Ovarian	Paclitaxel	^73^
miR-181a	PTEN	NSCLC	Paclitaxel, Cisplatin	^74^
miR-196a	MDR1/MRP1	NSCLC	Cisplatin	^75^
miR-200c	P-gp/ABCB1	Colorectal	Vincristineoxaliplatincisplatin 5-fluorouracilmitomycin C	^76^
miR-202	BAFF	Multiple myeloma	Bortezomib, Thalidomide, Dexamethasone	^77^
miR-217	PTEN	Breast	Tamoxifen, Etoposide, Lapatinib	^78^
miR-221/222	MRP1/ABCC1	Multiple myeloma	Melphalan	^79^
miR-508-5p	P-gp/ABCB1	Gastric	Vincristineadriamycincisplatin 5-fluorouracil	^80^
miR-519c	ABCG2	Colorectal	5-fluorouracil	^81^
miR-634	CCND1, GRB2, ERK2, RSK1, RSK2	Ovary	Cisplatin	^82^
miR-4689	KRAS, AKT1	NSCLC	EGFR inhibitors	^83^

## Conclusion

We know that the overdose of the antibiotics leads to drug resistance to the bacteria. Thus, the rapid cell division and high frequencies of mutations cause the natural selection of the resistant strains of these bacteria and survive in the presence of the certain drugs. Also, the human cancer cells with high proliferation rate are genetically unstable, so, the drug resistance could occur in a similar way. Interestingly, the studies approved that cancer cells which are smart, and resistance to the cellular stresses and agents have been created via altered mechanisms of the cell biology. The cancer drug resistance is a complex phenomenon. Thus; the combination therapy is the best option for drug resisted type of cancers. In this context, we reviewed different involved mechanisms in drug resistance and finally, we found the epigenetic drugs and synergy or an additive effect between established chemotherapeutic agents in combination with each other might provide a new strategy in drug resistance cancers. New studies suggested that cancer cells could sensitize to chemotherapeutic agents, via RNAi technique (such as miRNA), consequently with RNAi strategy (espcially siRNA) the chemotherapy drug resistance genes suppressed and limited the drug resistance in the resisted tumoral cells. Generally, there are two strategies in treatment with miRNA based therapy including miRNA replacement and miRNA masking. The replacement of tumor suppressor miRNA and suppression of oncomiRs can regulate cancerous cells by suppressing their target genes which are involved in cancer development especially cancer drug resistance.^84-88^ Also the combination of chemotherapy agents with RNAi strategy (siRNA or miRNA) might be a potetial therapy in the resisted tumoral cells.

## Ethical Issues

Not applicable.

## Conflict of Interest

The authors declare no conflict of interests.
